# Metabolomic analysis of the impact of red ginseng on equine physiology

**DOI:** 10.3389/fvets.2024.1425089

**Published:** 2024-09-30

**Authors:** Young Beom Kwak, Ilia Stambler, Hye Hyun Yoo, Jungho Yoon

**Affiliations:** ^1^Department of Pharmaceutical Engineering, Inje University, Gimhae, Republic of Korea; ^2^Department of Science, Technology and Society, Bar-Ilan University, Ramat Gan, Israel; ^3^Pharmacomicrobiomics Research Center and College of Pharmacy, Hanyang University, Ansan, Gyeonggi-Do, Republic of Korea; ^4^Equine Clinic, Jeju Stud Farm, Korea Racing Authority, Jeju-si, Jeju, Republic of Korea

**Keywords:** red ginseng, metabolomics, biomarkers, sphingolipid metabolism, glycerophospholipid metabolism

## Abstract

**Introduction:**

Red ginseng (RG), a traditional herbal remedy, has garnered attention owing to its diverse health benefits resulting from its complex composition. However, extensive research is needed to substantiate the efficacy of RG and understand the underlying mechanisms supporting these benefits. This study aimed to identify potential biomarkers and investigate the impact of RG on related metabolic pathways in horse plasma using liquid chromatography–mass spectrometry (LC–MS)-based metabolomics.

**Methods:**

Ten horses were divided into control and RG groups, with the latter administered RG at a dose of 600 mg⋅kg^−1^⋅day^−1^ for 3 weeks. Subsequently, the plasma samples were collected and analyzed using LC–MS. Multivariate statistical analysis, volcano plots, and feature-based molecular networking were employed.

**Results:**

The analysis identified 16 metabolites that substantially decreased and 21 metabolites that substantially increased following RG consumption. Among the identified metabolites were oleanolic acid, ursolic acid, and ginsenoside Rb1, which are known for their antioxidant and anti-inflammatory properties, as well as lipid species that influence sphingolipid and glycerophospholipid metabolism. Additionally, potential biomarkers, including major RG components, demonstrated distinct group clustering in principal component analysis and partial least squares-discriminant analysis, indicating their utility in assessing the physiological effects of RG consumption.

**Discussion:**

This study contributes to a comprehensive understanding of the effects of RG on health.

## 1 Introduction

Red ginseng (RG) is a medicinal herb traditionally used as a supplement for centuries in various treatments, owing to its composition of polyacetylenes, polysaccharides, sesquiterpenes, peptidoglycans, nitrogen-containing compounds, phenolic compounds, and vitamins (1). RG has been the subject of extensive scientific research due to its potential health benefits. The active components of RG exhibit diverse biological effects, including anti-inflammatory, antitumor, antidiabetic, antioxidant, and anti-stress activities in various tissues (2–5). These properties underscore the potential of RG as an alternative medicine for preventing and treating a range of diseases, including cardiovascular, metabolic, neurological, and hepatic disorders (2–5).

However, there are various perspectives on the precise mechanisms and metabolic pathways underlying the diverse effects of RG (6). To comprehensively understand these effects, a systematic investigation into the molecular mechanisms of its constituents is essential. Mass spectrometry offers a comprehensive and sensitive approach for substance detection, and liquid chromatography–mass spectrometry (LC–MS)-based metabolomics provides new insights into the efficacy of RG (7–12). A systematic analysis of metabolic responses of RG, along with a detailed examination of its mechanisms (13), is crucial to elucidate the synergistic effects of its different components, assess its safety, and gain a deeper understanding of complex molecular mechanisms of RG (14).

Current efforts to evaluate the health effects of RG often rely on studying changes in blood metabolites, predominantly using rodent models (1, 15). However, to understand benefits of RG in humans, it is crucial to consider interspecies specificity. Therefore, experimental evidence from diverse species is imperative to align these results with human outcomes (16).

Studying the effects of RG consumption in healthy horses and evaluating specific biomarkers can provide valuable information. First, it can validate the effects of RG on health. Second, it can assess individual physiological responses, enabling personalized health management (17). Third, specific biomarkers can be monitored to confirm the safety of long-term RG consumption and assess any potential side effects (18). Fourth, it can aid in the early prediction and prevention of potential health issues (19). Lastly, studying changes in specific biomarkers can elucidate the physiological effects of RG, contributing to a more comprehensive understanding of its mechanisms of action (20).

This study aimed to investigate, for the first time, the effect of RG consumption on horse plasma metabolites and to identify potential biomarkers associated with RG intake. Using LC–MS-based metabolomics, this study explored alterations in metabolic pathways and the potential physiological effects of RG. These findings suggest that specific metabolites, including ginsenosides, May serve as potential biomarkers to assess the effects of RG on health.

## 2 Materials and methods

### 2.1 Red ginseng consumption in horses

Ten-adult Thoroughbred horses were randomly divided into two groups of five horses each. All horses in the study were bred and supervised under the same conditions on a farm for a minimum of 1 year. The trial group received ground RG (600 mg⋅kg^−1^⋅day^−1^) mixed with molasses as a carrier every morning for 3 weeks, while the control group received only the carrier. All horses *ad libitum* access to food and water throughout the study period. In the third week, venipuncture was performed in the morning following overnight fasting. Twenty milliliters of blood were drawn into heparin tubes (BD Vacutainer®). Animal protocols were approved by the Institutional Animal Care and Use Committee of the Korea Racing Authority (KRA IACUC-2207-AEC-2207).

### 2.2 Data mining and preprocessing

The materials, sample preparation, and analysis methods are described in Supplementary material S1. The workflow chart for the study is shown in Supplementary Figure S1. Excel files (including features such as *m/z* values, retention times, and peak area information) for omics analysis, along with MGF format files for metabolite annotation and feature-based molecular networking (FBMN), were generated and exported from MZmine 3 (version 3.9.0). The data were calibrated using internal standards. Outliers were removed using a filter based on the QC function, and the dataset was preprocessed through log-transformation and Pareto-normalization in MetaboAnalyst 5.0 (21).

### 2.3 Peak annotation using *in silico* methods

Peak annotation was performed using SIRIUS version 5.8.3 software (22). MS1 and MS2 peak fragmentation patterns were interpreted with ZODIAC, CSI: FingerID, and CANOPUS using the ClassyFire and NPClassifier modules. All the datasets were used for tentative identification. For the potential identification of biomarkers, COSMIC, ZODIAC, and confidence scores were comprehensively considered. Candidates with a ZODIAC score of 50% or lower or a SIRIUS score of <50% without an accompanying ZODIAC score were excluded from the analysis. A comprehensive approach was employed to verify additional biomarkers using the Human Metabolome Database (HMDB, https://hmdb.ca/), mzCloud (https://www.mzcloud.org), and Lipid Maps (https://www.lipidmaps.org) databases, and a literature review. To facilitate pattern verification and data visualization, FBMN was employed (23). MGF files were uploaded to the Global Natural Product Social Molecular Networking (GNPS) platform (23).^1^ FBMN was conducted under the following conditions: minimum pair cosine score, 0.5; network topology, 10; and maximum connected component size, 100. The FBMN data were visualized and annotated using Cytoscape 3.10.1 software (San Diego, CA, United States).

### 2.4 Statistical and enrichment analysis

Principal component analysis (PCA) and partial least squares-discriminant analysis (PLS-DA) were employed to chemometrically visualize the discrimination between the RG and control groups and to identify the variables contributing to the separation between these groups using the MetaboAnalyst 5.0 platform.^2^ A volcano plot was used to display the results of the fold change and independent t-tests (*p* < 0.01). The Lipid Pathway Enrichment Analysis platform^3^ and pathway analysis in MetaboAnalyst were used for functional enrichment analysis of the identified metabolites. To assess the sensitivity and specificity of the potential biomarkers, receiver operating characteristic (ROC) analyses were performed using MetaboAnalyst 5.0.

## 3 Results

### 3.1 Metabolomics method validation

QC samples were generated by combining equal volumes of extracts from all samples and were processed and tested identically to that of the analyzed samples. During the instrumental analysis, each QC sample was subjected to both positive and negative ion mode detection. The total ion chromatography spectra of the QC samples exhibited well-defined peaks and a consistently uniform distribution under these detection conditions, validating the robustness of the system (Supplementary Figure S2).

### 3.2 Multivariate statistical analysis and metabolic profile changes

**Figure 1:**
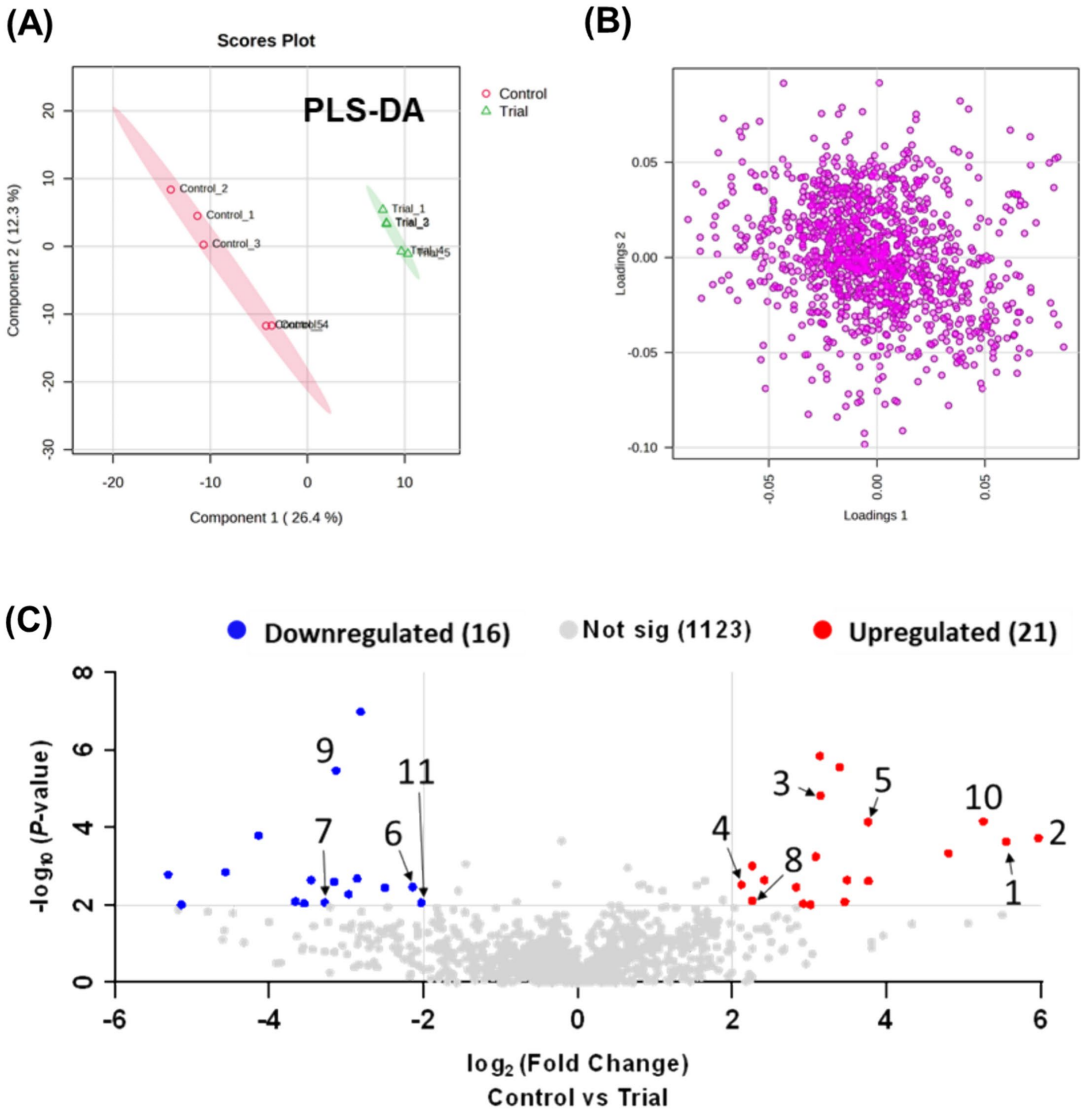
**(A)** Partial least squares-discriminant analysis plot based on LC-Q exactive data processed using Metaboanalyst 5.0, showing component 2 versus component 1. **(B)** Loading plot. **(C)** Volcano plot analysis.

Metabolic profiling and comprehensive analysis of metabolites between the RG and control groups using MZmine 3 detected 854 ions (positive mode) and 306 ions (negative mode) in the plasma samples. A total of 1,160 ions detected in both modes were integrated for statistical analysis. PCA was initially employed for an unsupervised comprehensive overview of the plasma samples. However, PCA did not reveal significant differences between the control and RG groups (data not shown). Subsequently, a supervised approach, PLS-DA, was employed to assess the differences between the groups. As shown in Figure 1A, the PLS-DA score plot revealed a clear distinction between the control and RG groups, suggesting substantial alterations in the metabolic profile of horse plasma following RG consumption (Figure 1B). Volcano plots were used to visualize the metabolites influencing the intergroup variations in PLS-DA. Comparing the RG-fed group with the control group, metabolites with log_2_ (fold change) values ≥2 and -log_10_ (*p*-value) values ≥2 were considered statistically significant. As a result, 16 metabolites exhibited a statistically significant decrease, whereas 21 metabolites showed a statistically significant increase in response to RG (Figure 1C). The red and blue dots indicate characteristic metabolites in the direction of comparison (RG consumption/control, adjusted *p*-value <0.01, fold change >2). Gray dots indicate the metabolites that did not differ between the two groups. Table 1 lists the numbers identified in the figure.

**Table 1:** Potential biomarkers associated with RG consumption in equine plasma (positive and negative mode).

No.	Mode	Tentative identification	Formula	Retention time	Precursor (*m/z*)	MS2 (*m/z*)	Trial/Control
1	Pos	Oleanolic acid	C_30_H_46_O_2_	7.58	439.3564	107.08, 133.10, 187.14	↑
2		Ursolic acid	C_28_H_36_O_15_	12.05	457.3669	439.35, 119.08, 147.11	↑
3		Ginsenoside Rb1	C_30_H_50_O_2_	18.66	425.3771	407.36, 109.10, 135.11	↑
4		SM(d16:1/17:0)	C_38_H_77_N_2_O_6_P	19.70	689.5584	184.07, 368.38, 124.99	↑
5		Phosphatidylcholine (18:3n6/18:2n6)	C_44_H_78_NO_8_P	22.22	802.5346	743.46, 619.46, 184.07	↑
6		N-ethyldocosanamide	C_24_H_49_NO	22.67	368.3883	102.09, 116.10, 130.12	↓
7		Cer 16:3;2O/16:1;(2OH)	C_32_H_57_NO_4_	22.74	520.4356	177.05, 149.05, 233.11	↓
8		PEG 25 cetostearyl ether	C_66_H_134_O_26_	23.80	1343.9276	664.45, 663.45, 383.14	↑
9		N,N-dibutylerucamide	C_30_H_59_NO	24.15	450.4664	130.15, 198.18, 156.13	↓
10	Neg	Octillol	C_30_H_52_O_5_	12.04	491.3745	473.36, 115.07, 375.29	↑
11		8(S)-hydroxyeicosatetraenoic acid	C_20_H_32_O_3_	14.00	319.228	179.10, 301.21, 257.22	↓

↑ indicates increase; ↓ indicates decrease. *p* < 0.01.

### 3.3 Identification of metabolites

We annotated the significantly altered metabolites, and among the annotated metabolites, 11 out of 37 (approximately 29%) were identified based on MS1 and MS2 fragmentation data (Table 1). The identified metabolites included three major components–oleanolic acid, ursolic acid, and ginsenoside Rb1–as well as various lipid components. For the 26 peaks for which significant identification results were not obtained using the MS1 and MS2 spectra, the substances predicted using MS1 information were verified through the HMDB (Supplementary material S2, molecular weight tolerance < ±10 ppm). Among the potential metabolites identified, representative substances included phosphatidylethanolamine, oxidized ceramide, and cardiolipin.

### 3.4 ClassyFire information

**Figure 2:**
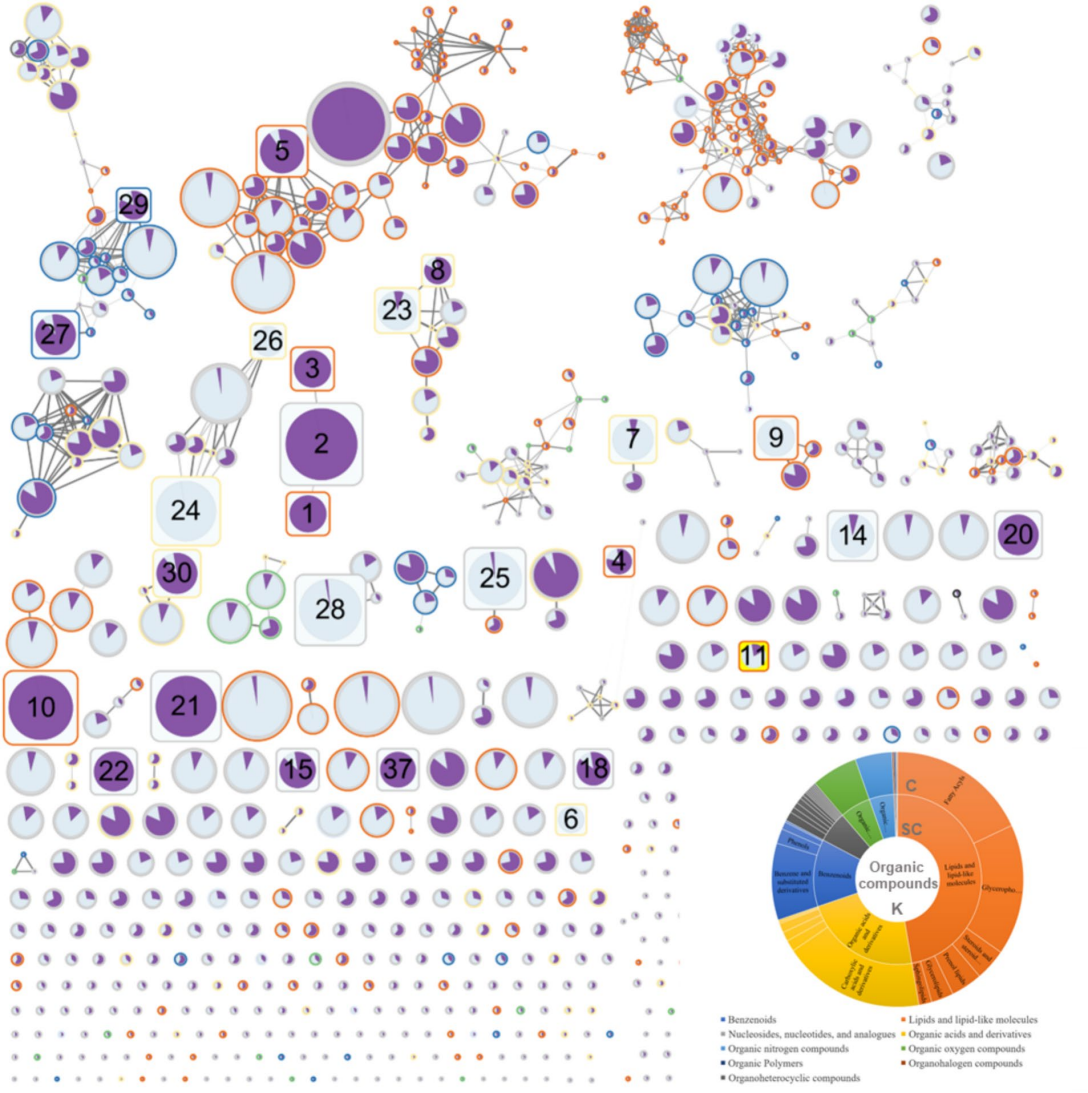
Results of feature-based molecular networking between the red ginseng (RG) consumption group and the control group.

Using ClassyFire to categorize the compounds identified in equine plasma, the majority of the compounds were summarized according to the kingdom (K), superclass (SC), and class (C) levels. Specifically, in the SC classification, most compounds were lipids and lipid-like molecules (47.2%), organic acids and their derivatives (22.5%), and benzenoids (12.8%) (Figure 2). FBMN allowed the visualization of connections between compounds based on the integration of ClassyFire information and the significance of peaks (Figure 2).

### 3.5 Feature-based molecular networking analysis

The FBMN analysis generated 780 nodes (Figure 2). In comparison, 1,160 peaks were obtained during the data mining process. The smaller number of nodes (780) was attributed to the use of the data-dependent acquisition (DDA) mode in the analysis. The DDA approach relies on MS1 data to induce a limited MS2, providing the advantage of obtaining a more reliable MS2 data for the target MS1. However, it failed to acquire all MS2 information for trace metabolites, which is crucial for FBMN analysis. Therefore, 11 of the 37 peaks that exhibited significant changes (as indicated in Table 1 and Supplementary material S2, with numbers 12, 13, 16, 17, 19, 31, 32, 33, 34, 35, and 36) were not represented as nodes. The FBMN results demonstrated that compounds with similar backbones, such as oleanolic acid, ursolic acid, and ginsenoside Rb1, were interconnected. These compounds were not detected in the control group, indicating that RG as their source. Additionally, compound 23 (Supplementary material S2), with structural similarity to compound 8, identified with high confidence (Table 1, PEG 25 cetostearyl ether), suggests the possibility of it being phosphatidylinositol-3,4,5-trisphosphate among the predicted compound candidates (momorcharaside B, notoginsenoside R9, ginsenoside M7cd, and phosphatidylinositol-3,4,5-trisphosphate). Additionally, the fold-change feature values in the RG consumption group compared to that of the control group were used to determine node sizes by incorporating omics information. The color (from white to black) and thickness of the node edges are proportionally represented by cosine values. The average intensity values between the groups were visualized within nodes using a pie chart, with the RG consumption group represented in purple and the control group represented in sky blue. The CANOPUS analysis results, conducted using ClassyFire on data mined from MZmine3, were organized into a sunburst plot. Individual peak information is highlighted by node border colors (Figure 2). Finally, nodes representing characteristic metabolites (RG-fed/control, adjusted *p*-value <0.01, fold change >2 times) are denoted by rounded rectangles, and specific node numbers can be cross-referenced with the information in Table 1.

### 3.6 Enrichment analysis

**Figure 3:**
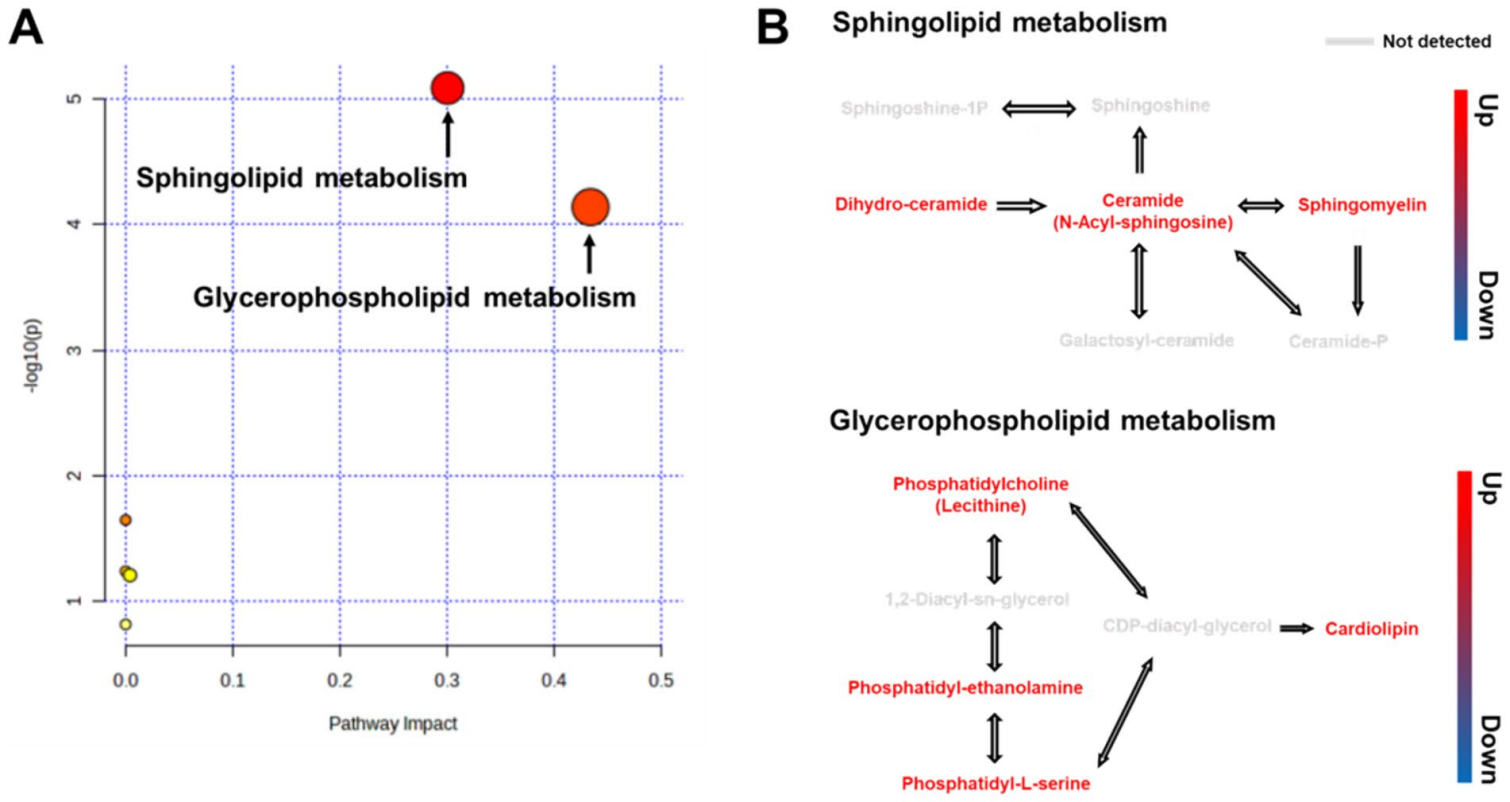
**(A)** Overview of pathway analysis for all metabolic pathways (Kyoto Encyclopedia of Genes and Genomes) for RG-fed horses. All matched pathways are classified by *p*-values (y-axis) from pathway enrichment analysis and pathway impact values (x-axis) from pathway topology analysis. Node size represents the impact values, while node colors indicate different *p*-values. **(B)** Pathway map of differential metabolites between RG-fed and control groups.

To analyze the metabolic pathways associated with the identified biomarkers after RG consumption, we used LIPEA and MetaboAnalyst 5.0 platforms. A total of 22 pathways were identified. RG administration had the greatest influence on sphingolipid (SL) and glycerophospholipid (GPL) metabolic pathways (Figure 3A). RG affected lipid metabolism by upregulating three metabolites (dihydroceramide, ceramide, and sphingomyelin) in the SL pathway and four metabolites (phosphatidylcholine, phosphatidyl-ethanolamine, phosphatidyl-L-serine, and cardiolipin) in the GPL pathway (Figure 3B).

## 4 Discussion

RG components produce diverse effects on the body, providing benefits in cardiovascular, hepatic, neurological, and metabolic disorders (2–5, 24, 25). However, the precise mechanisms and metabolic pathways underlying the effects of RG remain unclear. In this study, we aimed to identify biomarkers associated with RG administration and explore the biological pathways related to these biomarkers using systems biology approaches.

While numerous studies have investigated the health benefits of RG using rodent models (26–29), it is crucial to understand the mechanisms of action of RG components in other species. Extrapolating results from different species to humans remains uncertain. RG is commonly used as a feed additive and alternative medicine for horses to enhance performance, promote growth, and prevent diseases (30, 31). Over the past 5 years, quality test results for feed additives used in racehorses in Jeju, Korea, revealed that RG has been used extensively, accounting for 34.7% (76 out of 219 total feed additive inspections), indicating its frequent used in the equine industry. However, scientific evidence concerning its effects in horses is limited. This underscores the need to obtain experimental evidence in diverse species including horses (16). We observed changes in the plasma metabolites of horses fed RG to enhance our understanding of the impact of RG on their health. Furthermore, we statistically analyzed and visualized the plasma metabolites using PLS-DA and FBMN. Additionally, we identified metabolites potentially contributing to the observed changes through *in silico* methods and database analyses. The PLS-DA score plot clearly differentiated the blood samples from the control and RG groups. We also confirmed that the major representative components of RG and the plasma metabolites primarily contributed to these differences and were predominantly associated with lipid metabolism.

The potentially identified compounds–oleanolic acid, ursolic acid, and ginsenoside Rb1–are well-known representative components of RG (29, 32). Oleanolic acid and ursolic acid are structurally similar triterpenoid compounds known to inhibit inflammatory responses and reduce oxidative stress, thereby contributing to the mitigation of various chronic diseases. However, owing to structural differences, they modulate cellular signaling pathways differently, with ursolic acid reportedly exhibiting stronger anti-inflammatory effects than oleanolic acid (33, 34). Ginsenoside Rb1 exerts neuroprotective effects, improves brain function, and slows the progression of neurodegenerative diseases. It reduces stress, aids in fatigue recovery, and enhances overall physical vitality. Additionally, it contributes to blood pressure regulation and vascular function improvement, potentially reducing the risk of cardiovascular diseases (35–37).

Rb1 is the major ginsenoside in the plasma of horses (29). The findings of the current study emphasize that Rb1 is the predominant component in blood following RG consumption.

Changes in plasma metabolite levels due to RG consumption have been reported in various studies. A study administering RG daily to healthy mice for 6 weeks (3 mL, 300 mg/kg) observed an increase in plasma lipid metabolites (lysophosphatidylcholine, SL) (1). Similarly, a study conducted in a mouse model of Alzheimer’s disease showed significant changes in lipid metabolites (38). These findings strongly suggested that RG is primarily associated with lipid metabolism, reinforcing the reliability of the results obtained in this study.

We identified two significantly altered pathways following RG consumption: SL and GPL metabolism. SL and GPL are distinct lipid classes, and various lipid species within these classes participate in diverse cellular functions, including cell proliferation, signaling cascades, and apoptosis (39). The SL metabolic pathway begins with sphingosine and subsequently generates various SLs, such as ceramide and sphingomyelin. Ceramide plays a crucial role in apoptosis and cellular stress responses (40). The primary component of RG, ginsenosides, exhibit anti-inflammatory and antioxidant effects, which May contribute to the regulation of ceramide synthesis and degradation within the SL metabolic pathway. For example, ginsenosides can reduce cellular stress and promote cell survival (41, 42). GPL, consisting of a glycerol backbone attached to phosphate and fatty acids, are key components of the phospholipid bilayer. These lipids maintain cell membrane fluidity and play essential roles as secondary messengers in signaling pathways (43). RG consumption improves membrane fluidity and structural stability by regulating GPL metabolism (44). Some studies have suggested that RG consumption May positively influence blood lipid profiles and potentially improve cardiovascular health (45, 46).

Recent evidence suggests that SL and GPL pathways mutually regulate their biosynthesis, and alterations in these interactions can contribute to lipotoxicity-related impairments in various organs (47). RG exhibits adaptogenic properties in improving immunological and neurological diseases, with SL and GPL metabolism implicated in these process (48, 49). The results of this study demonstrated that RG administration in horses induced SL and GPL changes, with no relevant side effects. This strengthens our understanding of the diverse physiological effects of RG through the SL and GPL metabolic pathways.

Some studies have highlighted the role of ginsenosides in the changes in lipid composition resulting from RG consumption. Compound K plays a crucial role in regulating the biological functions of cells by increasing the expression of SL and ceramides (50). Additionally, Rb1 stimulates skin wound healing through a sphingolipid-1-phosphate-dependent mechanism and inhibits hyperlipidemia by regulating the synthesis and breakdown of phosphatidylcholine in GPL metabolism while also modulating the gut microbiota (15, 51).

However, understanding the intricate mechanisms underlying the effects of RG remains challenging, even with the evidence of lipid pathway alterations presented in this study. The effects of RG, such as the modulation of intestinal microbiota by Rb1, are influenced by various internal and external factors, resulting in enhancement or reduction of their effects. Gut microbes are closely linked to host metabolism, and SL produced by gut bacteria are subsequently transported to internal organs, affecting host lipid metabolism (52). Furthermore, RG modulates the diversity of the gut microbiota that can regulate the transformation of ginsenosides (52). Therefore, it can be inferred that RG, SL, and gut microbes intricately and reciprocally influence each other, potentially affecting the pathogenesis of various diseases. Comprehensive research on the relationship between gut microbes and RG is necessary for future studies.

This study evaluated the effects of RG supplementation in horses by analyzing both the metabolic pathways and physiological effects. However, several factors must be considered when interpreting these results. First, although RG has been used as an herbal medicine in Eastern countries for thousands of years (53), there are still no clear standards regarding its dosage and duration of administration. Previous studies in humans and rodents have used doses ranging from 100 to 600 mg/kg over periods ranging from 1 d to 27 months (54, 55). Considering these data and the resources available for our experiments, we administered RG at a dose of 600 mg/kg for 3 weeks. Furthermore, large animals like equines require more resources, cost, and specialized facilities compared to other species and considering the difficulty in collecting samples, conducting experiments involving numerous animals is challenging. Therefore, we conducted the experiment using 10 horses, which May appear relatively few compared to experiments using other animal models, such as mice. These physical and methodological limitations have contributed to the scarcity of *in vivo* horse data. Therefore, the data from this study warrants future large-scale experiments with rodents to build upon these findings.

## 5 Potential biomarkers

We assessed whether the major components of RG detected in plasma and the significantly altered plasma metabolites could potentially serve as biomarkers of RG intake. The evaluated 37 components had variable importance in projection values exceeding 1, and the area under the curve (AUC) values in the ROC analysis were 0.88 or higher (Supplementary Figure S3). PCA and PLS-DA of the 37 components revealed clear distinctions between the groups, and the heatmap results further confirmed group clustering. This suggests that these components could potentially be used as biomarkers in the future.

## 6 Conclusion

This study demonstrated significant alterations in the plasma metabolites of horses following RG consumption, particularly affecting lipid metabolism. The identified compounds, including oleanolic acid, ursolic acid, and ginsenoside Rb1–known for their antioxidant and anti-inflammatory properties–emerged as potential biomarkers for RG intake. This study provides insights into the metabolic effects of RG and contributes to a broader understanding of its potential health benefits. Additionally, this study serves as a foundation for tailored health management and safety assessments associated with long-term RG consumption.

## Data Availability

The original contributions presented in the study are included in the article/Supplementary material, further inquiries can be directed to the corresponding author/s.

## Footnotes

^1^ https://gnps.ucsd.edu/ProteoSAFe/static/gnps-splash.jsp

^2^ https://www.metaboanalyst.ca

^3^LIPEA, https://hyperlipea.org
