# Supplementary material for: Metabolomic analysis of the impact of red ginseng on equine physiology
Source: Front Vet Sci. 2024 Sep 30;11:1425089. doi: 10.3389/fvets.2024.1425089 (PMC11471734; doi:10.3389/fvets.2024.1425089)
Supplement: Supplementary file 1 [file Data_Sheet_1.docx]

**Supplementary data 1**

**Materials**

The study utilized Korean red Ginseng Powder Gold produced by the Pocheon Ginseng Farming Association in the Republic of Korea. This product had a ginsenoside content of 19.65 mg/g, including ginsenosides, Rg1, Rb1, and Rg3. High-performance liquid chromatography (HPLC)-grade acetonitrile (ACN) and HPLC-grade deionized water (DW) were procured from J.T. Baker (Phillipsburg, NJ, USA). Formic acid (FA) was obtained from Junsei Chem (Chou-ku, Japan).

**Sample prepration**

The quality control (QC) sample was prepared by pooling all the specimens. The collected blood was subjected to centrifugation at 3000 rpm to separate the plasma layer. The plasma sample (100 μL) was subjected to deproteinization using 300 μL of ACN containing 100 ng/mL phenacetin and althiazide (internal standard, IS). The sample was vigorously vortex-mixed and then centrifuged at 13,200 rpm for 5 min. The resulting supernatant was transferred to LC vials and a 2 μL aliquot was injected into the UHPLC- Q-Exactive analysis system.

**LC-MS analysis of plasma**

Chromatographic separation of the metabolites was performed on a Thermo UHPLC system equipped with a Phenomenex LunaOmega column (1.6 μm, 2.1 × 100 mm; Phenomenex, Aschaffenburg, Germany). The mobile phases consisted of 5 mM ammonium formate (pH 3.0) in DW (solvent A) and 0.1% FA in ACN (solvent B). The solvent gradient was composed as follows: The B solvent was held at 30% for the initial 5 min, followed by a linear increase to 100% B solvent from the 5 to the 23 min. At 23.1 min, the B solvent was reverted to 30% and maintained up to the 25 min for equilibrating the systems. The sample injection volume was 2 μL, and the ﬂow rate was set to 0.25 mL/min. The column temperature was maintained at 40°C. During the period of analysis, all these samples were stored at 4°C. Mass spectrometric data were acquired using a Thermo Q-Exactive HF-X mass spectrometer equipped with an electrospray ionization (ESI) source operating in both positive and negative ion modes. The optimized conditions were as follows: Heater temperature at 425°C, capillary temperature at 320°C, sheath gas flow rate at 50 arb, Aux gas flow rate at 10 arb, ionization voltage at 3.5 kV in the positive mode and -4 kV in the negative mode, respectively. Normalized collision energy ranged from 20 to 50 V for MS/MS. The full MS resolution was set at 60,000, and the MS/MS resolution at 15,000. Data acquisition utilized the Data Dependent Acquisition (DDA) mode, scanning across a mass range of 100–1500 Da.

**
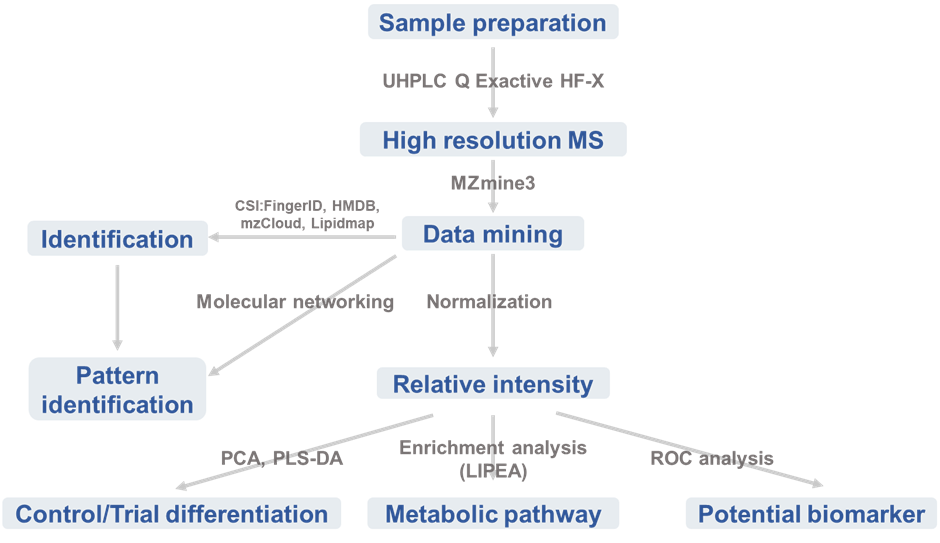
**

**Figure S1.** The workflow of the plasma screening method used in this study.

**
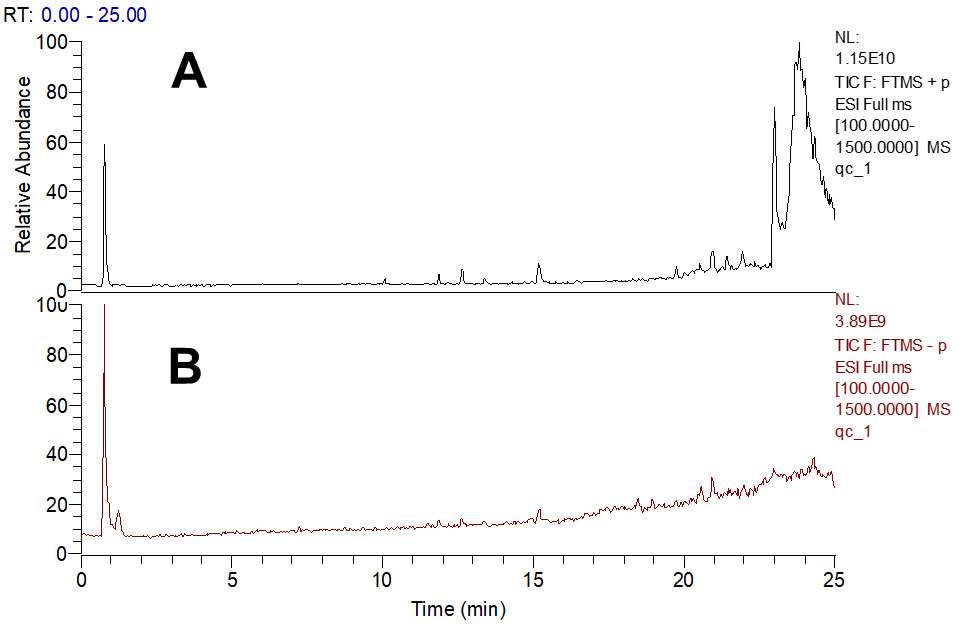
**

**Figure S2.** Chromatogram of QC **A.** total ion chromatogram (ESI +) **B.** total ion chromatogram (ESI -).

**
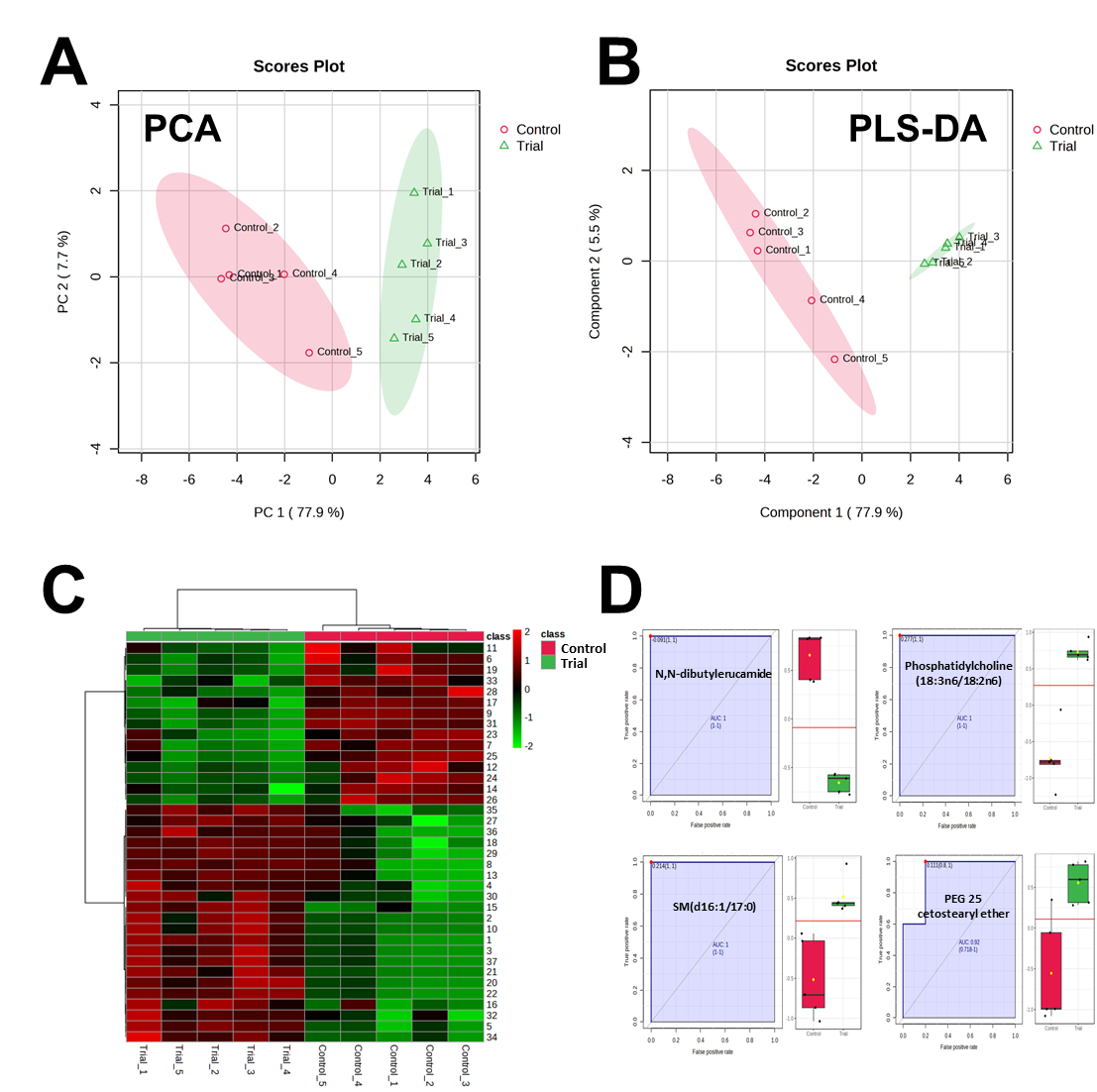
**

**Figure S3.** Potential biomarker analysis. The results of (A) Principal component analysis (PCA), (B) Partial least squares-discriminant analysis (PLS-DA), (C) Hierarchical clustering heatmaps, (D) Receiver operating characteristic curve (ROC) analysis between the red ginseng (RG) consumption and control group. 37 characteristic metabolites (RG consumption/control, adjusted *P*-value < 0.01, fold change > 2 times) assessed based on their area values.
